# Mitral Annular and Coronary Artery Calcification Are Associated with Mortality in HIV-Infected Individuals

**DOI:** 10.1371/journal.pone.0130592

**Published:** 2015-07-01

**Authors:** David C. Lange, David Glidden, Eric A. Secemsky, Karen Ordovas, Steven G. Deeks, Jeffrey N. Martin, Ann F. Bolger, Priscilla Y. Hsue

**Affiliations:** 1 Division of Cardiology, Department of Medicine, Cedars-Sinai Medical Center, Los Angeles, California, United States of America; 2 Department of Epidemiology & Biostatistics, University of California, San Francisco, San Francisco, California, United States of America; 3 Division of Cardiology, Department of Medicine, Massachusetts General Hospital, Boston, Massachusetts, United States of America; 4 Department of Radiology, University of California, San Francisco, San Francisco, California, United States of America; 5 Division of HIV/AIDS Positive Health Program, San Francisco General Hospital, University of California, San Francisco, San Francisco, California, United States of America; 6 Department of Epidemiology and Biostatistics, San Francisco General Hospital, University of California, San Francisco, California, United States of America; 7 Division of Cardiology, Department of Medicine, San Francisco General Hospital, University of California, San Francisco, California, United States of America; University of Pittsburgh Center for Vaccine Research, UNITED STATES

## Abstract

**Background:**

HIV infection increases cardiovascular risk. Coronary artery calcification (CAC) and mitral annular calcification (MAC) identify patients at risk for cardiovascular disease (CVD). The purpose of this study was to examine the association between MAC, CAC and mortality in HIV-infected individuals.

**Methods and Results:**

We studied 152 asymptomatic HIV-infected individuals with transthoracic echocardiography (TTE) and computed tomography (CT). MAC was identified on TTE using standardized criteria. Presence of CAC, CAC score and CAC percentiles were determined using the modified Agatston criteria. Mortality data was obtained from the Social Security and National Death Indices (SSDI/NDI). The median age was 49 years; 87% were male. The median duration of HIV was 16 years; 84% took antiretroviral therapy; 64% had an undetectable viral load. CVD risk factors included hypertension (35%), smoking (62%) and dyslipidemia (35%). Twenty-five percent of individuals had MAC, and 42% had CAC. Over a median follow-up of 8 years, 11 subjects died. Subjects with CAC had significantly higher mortality compared to those with MAC only or no MAC. The Harrell’s C-statistic of CAC was 0.66 and increased to 0.75 when MAC was added (p = 0.05). MAC, prior CVD, age and HIV viral load were independently associated with higher age- and gender-adjusted CAC percentiles in an adjusted model (p < 0.05 for all).

**Conclusion:**

In HIV patients, the presence of MAC, traditional risk factors and HIV viral load were independently associated with CAC. Presence of CAC and MAC may be useful in identifying HIV-infected individuals at higher risk for death.

## Background

Approximately 1.3 million HIV-positive adults are living in the United States and 34 million HIV-infected individuals are living world-wide [1]. Since the advent of highly active antiretroviral therapy (HAART) in the mid to late 1990s, HIV-associated morbidity and mortality have decreased substantially [2]. However, despite these advances in treatment, HIV-infected individuals continue have a shorter life expectancy when compared to uninfected controls [3,4]. In the United States, the annual number of HIV-associated deaths fell from greater than 50,000 per year to less than 25,000 per year between 1993 and 2000, but has remained relatively unchanged since this time [5]. This reduced life expectancy is thought to be largely secondary to non-AIDS conditions, which now account for the majority of deaths in HIV [6]. Of particular concern, HIV-infected patients are at increased risk for cardiovascular disease, though the mechanism is not completely understood [7–9]. HIV-infected patients have significantly higher rates of myocardial infarction compared to controls, even in the setting of treated and suppressed HIV disease [10]. In addition, individuals with HIV who are hospitalized for myocardial infarction have higher in-hospital mortality as compared to uninfected individuals [11]. HIV patients also have higher rates of coronary artery calcification and systemic arterial calcification than non-infected controls, which may reflect accelerated vascular aging [12]. Finally, our group previously reported that HIV-infected individuals have substantially higher rates of sudden cardiac death in comparison to uninfected individuals [13].

Mitral annular calcification (MAC) is a degenerative process involving the mitral valve annulus, and is typically associated with older age [14]. MAC has been described in the setting of various disease processes including systemic hypertension, aortic stenosis, hypertrophic obstructive cardiomyopathy, mitral valve prolapse, and diseases of abnormal calcium and phosphorus metabolism such as chronic kidney disease (CKD) or secondary hyperparathyroidism [14]. The presence of MAC closely correlates with systemic atherosclerosis [15,16], as well as the prevalence and severity of coronary artery disease [14,17,18]. MAC is an independent predictor of major cardiovascular events and mortality [19–26]. While these associations have been described in the general population, the clinical significance of MAC among HIV-infected individuals remains unclear.

Coronary artery calcification (CAC), identified on electron-beam computed tomography (EBCT), is an established technique for identifying patients with, or at risk for coronary artery disease [27]. The presence of CAC has proven to be predictive of cardiovascular disease, cardiovascular mortality, and all-cause mortality in both symptomatic and asymptomatic individuals [28–30]. Conversely, the absence of CAC on EBCT predicts excellent 10-year survival [31]. The purpose of this study was to examine the association between MAC and CAC, and to characterize their predictive value for mortality among asymptomatic HIV-infected individuals.

## Methods

### Study Design

We studied 152 HIV-infected individuals who underwent both Transthoracic Echocardiogram (TTE), and CAC Protocol External Beam Computed Tomography (CT) study between 11/1/2004 and 11/1/2012. The HIV-infected individuals were enrolled in an outpatient HIV-cohort in San Francisco called the Study of the Consequences of the Protease Inhibitor Era (SCOPE). Patients were seen in follow-up as part of the SCOPE study every four months.

### Patient Selection

The SCOPE cohort included patients who can be categorized as: (1) untreated HIV-infected patients, including a subset of individuals with undetectable HIV RNA levels, commonly referred to as elite controllers, (2) patients on HAART who have detectable viremia, and (3) treated patients who have achieved full virologic suppression. SCOPE patients are followed longitudinally and are well characterized with regards to their HIV status, metabolic profiles and cardiovascular risk factors. All patients who had at least one TTE and one CAC-protocol CT scan between 11/1/2004 and 11/1/2012 were included in the initial analysis. Patients were not preselected for cardiovascular risk factors or symptoms. Exclusion criteria included poor study quality that precluded the interpretation of any one of the aforementioned studies and individuals who could not obtain a CT scan due to renal disease (defined as estimated glomerular filtration rate < 60 mL/min), atrial fibrillation, or a history of prior CT scan contrast allergy. The University of California, San Francisco Committee on Human Research approved this study, and all patients provided written informed consent prior to enrollment.

### Risk Factor Assessment

At the time of enrollment, each patient completed a standardized questionnaire, and a detailed interview to compile sociodemographic characteristics, HIV disease history, presence of co-morbid conditions, health-related behaviors, medication exposures, and family history. Additionally, fasting serum measurements of glucose, HDL cholesterol, LDL cholesterol, total cholesterol, triglycerides, lipoprotein (a), high-sensitivity C-reactive protein, creatinine, CD4 count, and HIV viral load were obtained at the clinical laboratories of San Francisco General Hospital. Standardized waist and hip measurements were obtained, and each patient was assessed for changes in fat distribution.

### MAC Measurements

**Fig 1:**
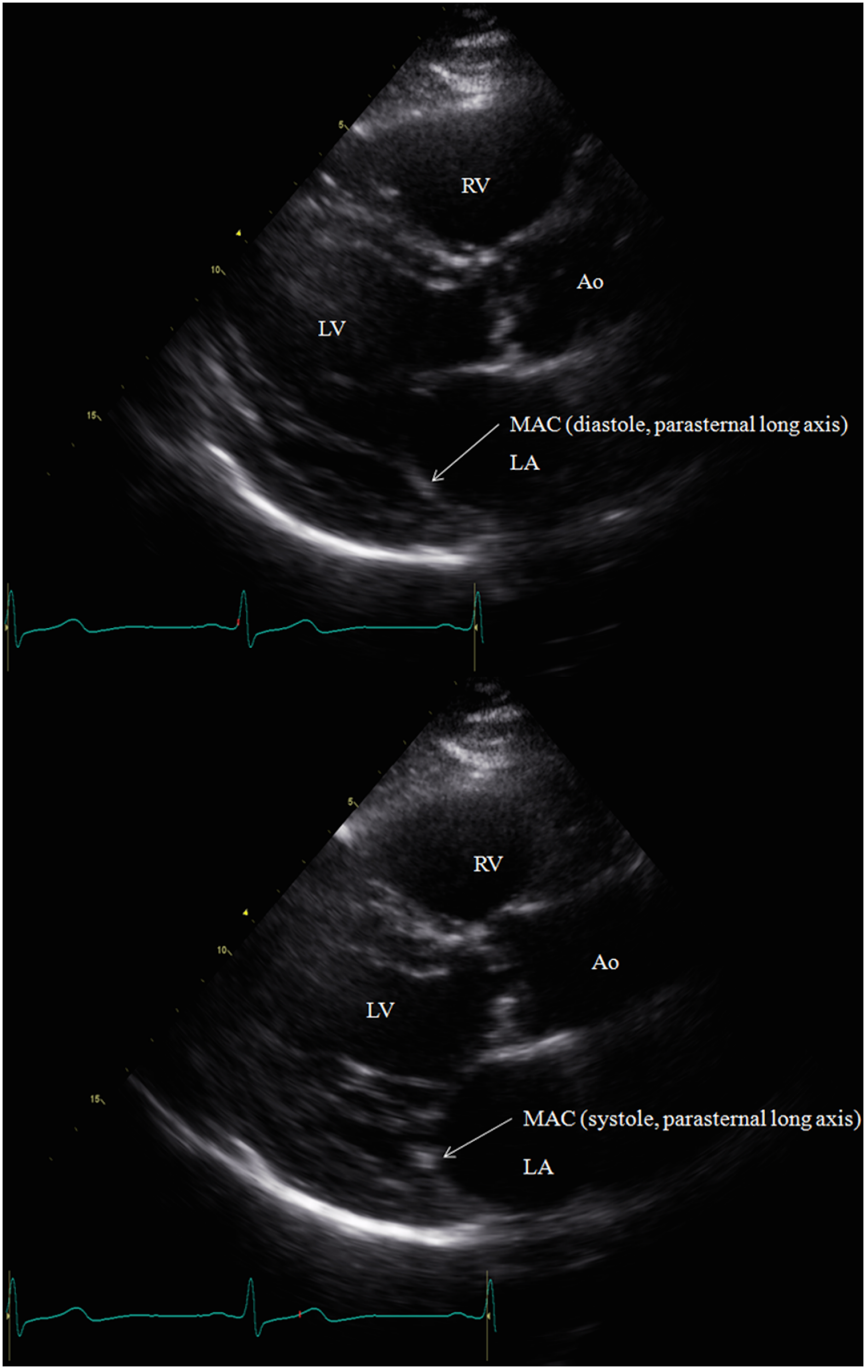
(A-B). MAC was defined as an echodense area visualized throughout systole and diastole, distinguishable from the posterior mitral valve leaflet, located anterior and parallel to the posterior left ventricular wall, seen here in the parasternal long axis view during diastole (A) and systole (B). Abbreviations: Right Ventricle (RV), Left Ventricle (LV), Aorta (Ao), Mitral Annular Calcification (MAC), Left Atrium (LA).

**Fig 2:**
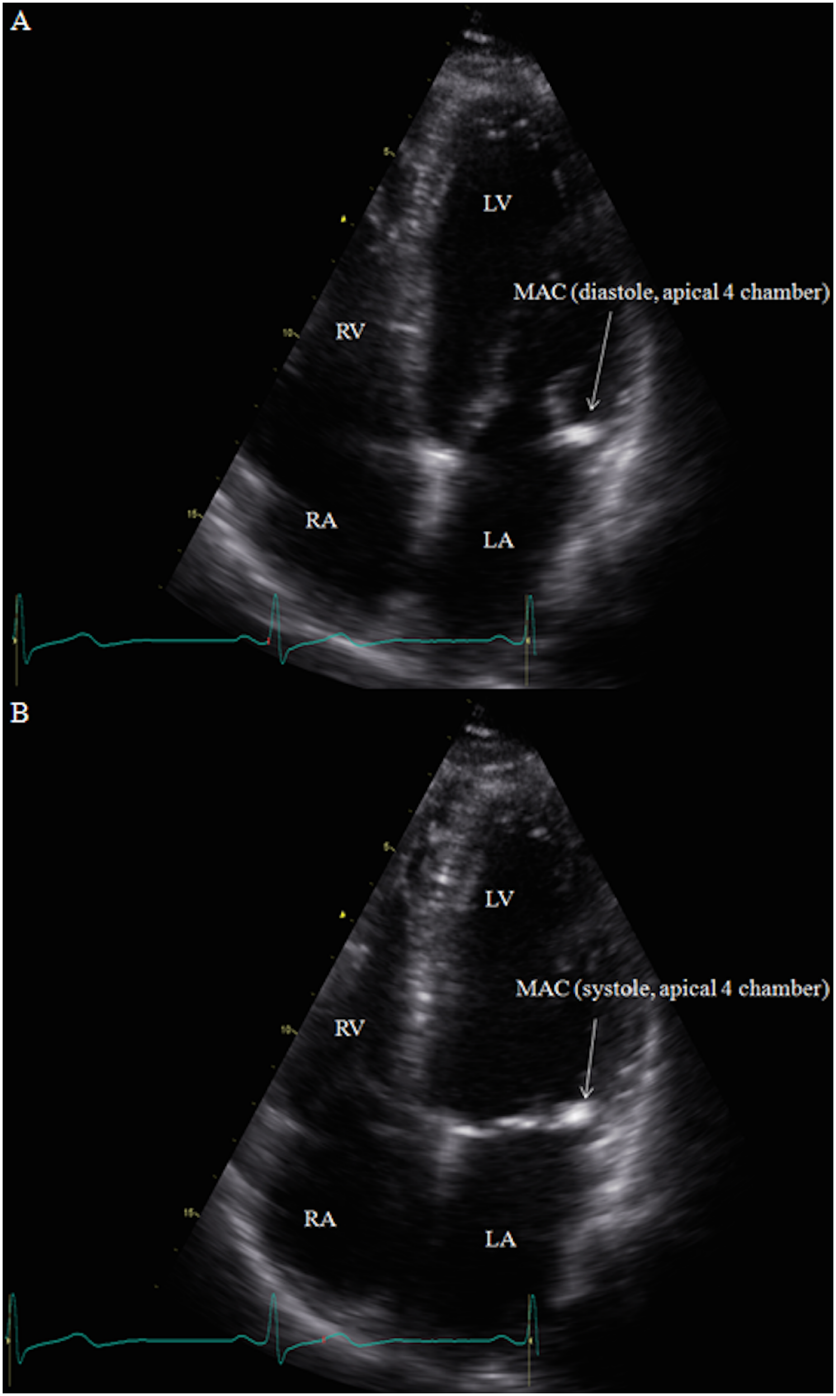
(A-B): MAC was defined as an echodense area visualized throughout systole and diastole, distinguishable from the posterior mitral valve leaflet, located anterior and parallel to the posterior left ventricular wall, seen here in the apical four chamber view during diastole (A) and systole (B). Abbreviations: Right Ventricle (RV), Left Ventricle (LV), Mitral Annular Calcification (MAC), Left Atrium (LA), Right Atrium (RA).

**Fig 3:**
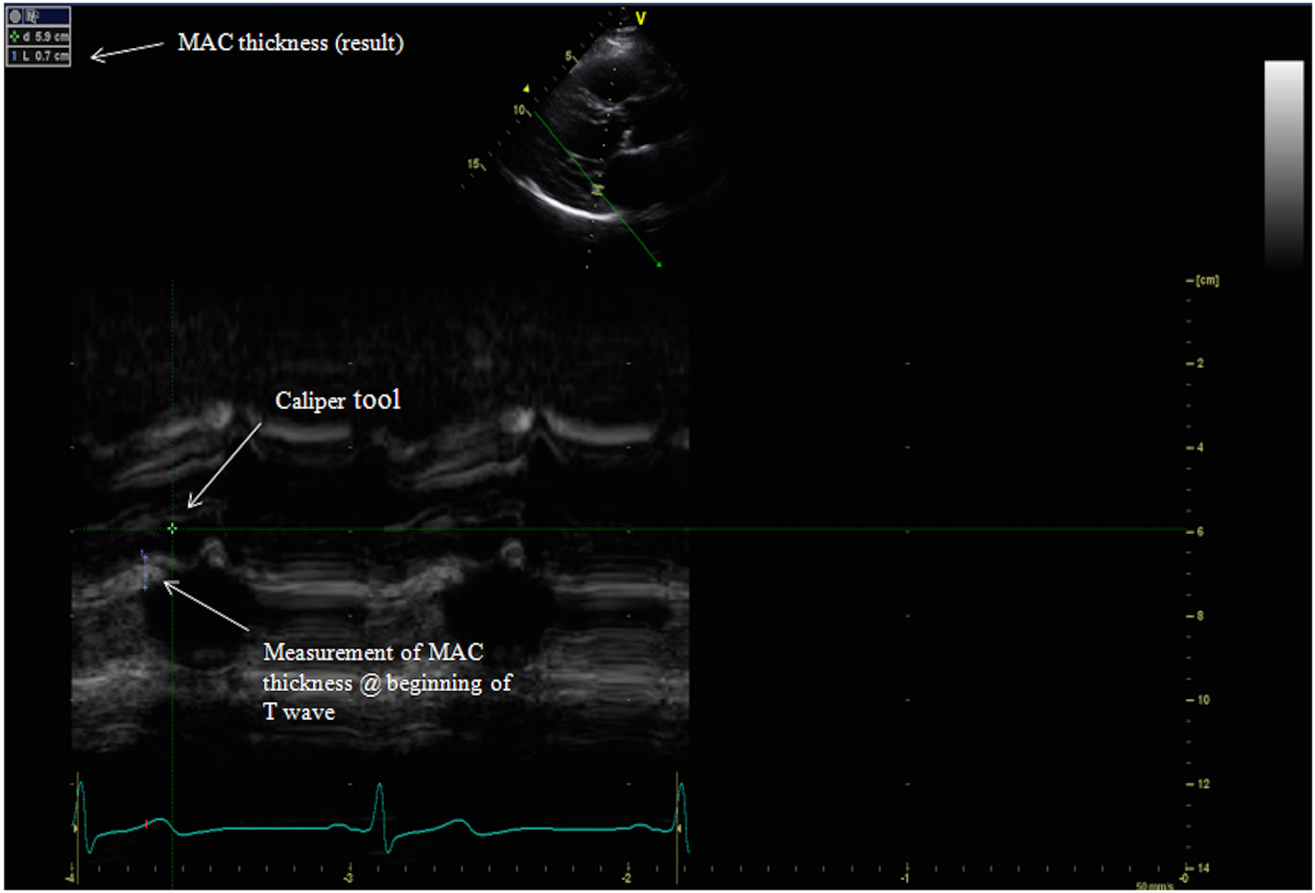
M-mode measurement of MAC in the parasternal long axis view. Abbreviations: Mitral Annular Calcification (MAC).

**Fig 4:**
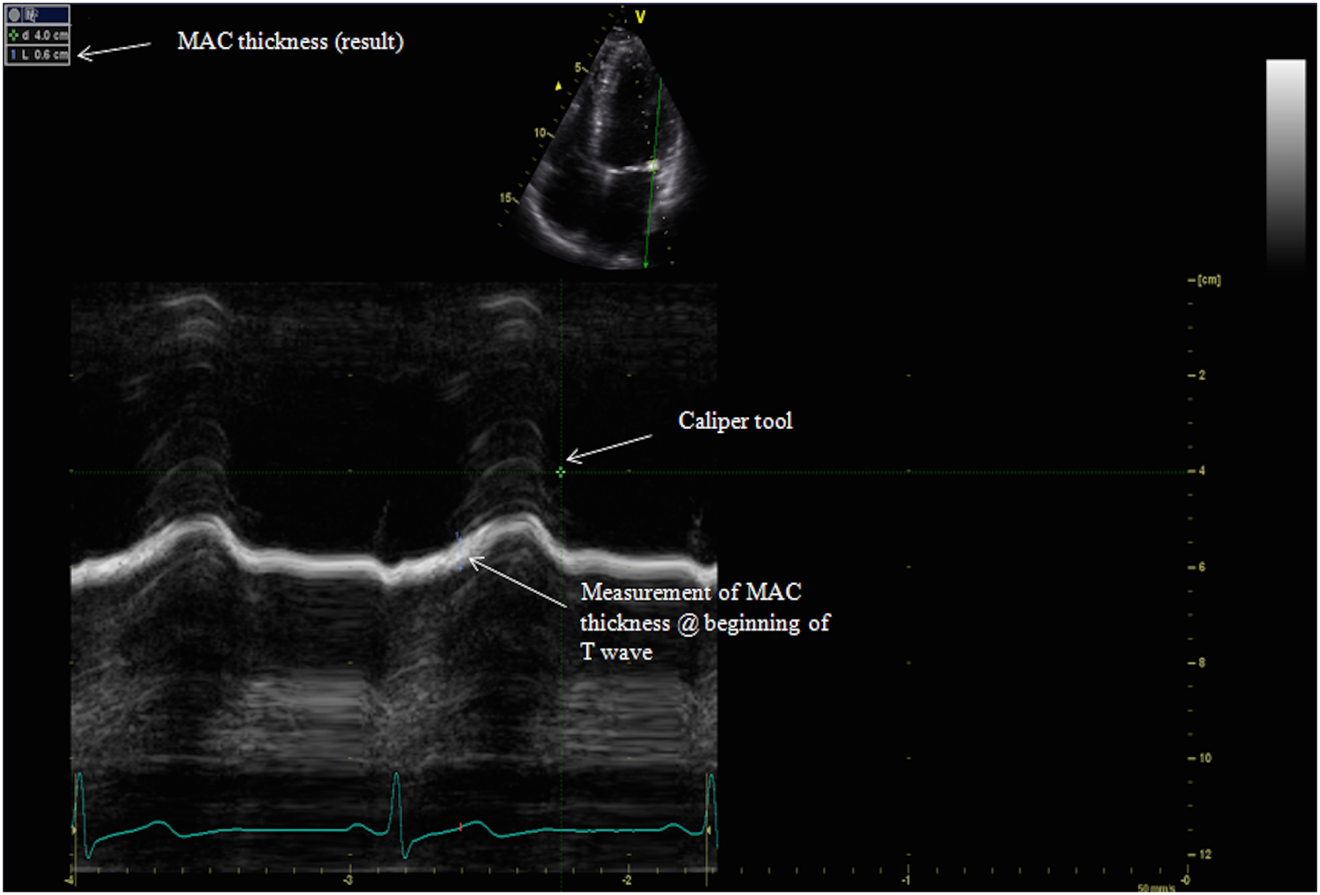
M-mode measurement of MAC in the apical four chamber view. MAC—Mitral annular calcification; cm—centimeters. Abbreviations: Mitral Annular Calcification (MAC).

Standard two-dimensional (2D) echocardiography recordings were obtained in the parasternal, apical and subcostal views. M-mode recordings were performed in the parasternal long axis and apical four chamber views (GE Vivid 7, GE Healthcare, Milwaukee, Wisconsin). Raw data images were all interpreted off line (GE ECHOPac PC software) by a cardiologist (D.L.) to determine the presence or absence of MAC. The cardiologist interpreting the echocardiograms was blinded to all clinical information about the subjects. MAC was defined as the presence of an abnormally echo-dense area visualized throughout systole and diastole, distinguishable from the posterior mitral valve leaflet, located anterior and parallel to the posterior left ventricular wall, and visualized in at least two views (e.g. parasternal long axis view and apical four chamber view) (Fig 1A and 1B;Fig 2A and 2B). These diagnostic criteria are consistent with those used in previous studies [15,23,26]. If MAC was present using these criteria, three measurements from consecutive beats were taken in the parasternal long axis and apical four chamber views using the caliper tool, following a pre-specified protocol (Figs 3 and 4).

### Coronary Calcium Measurements

**Fig 5:**
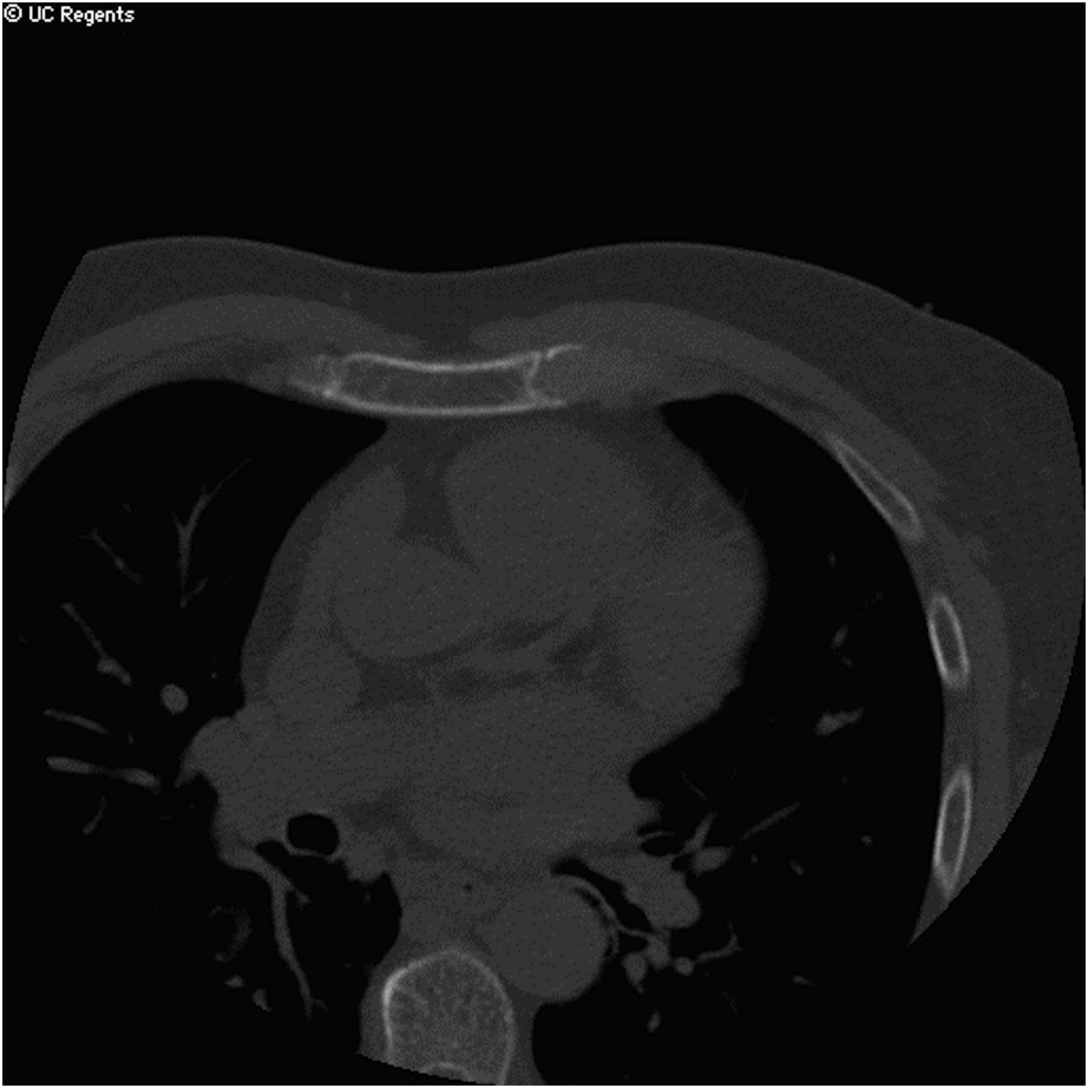
CAC EBCT of a subject with CAC score of 0. Abbreviations: Coronary artery calcium (CAC), External beam computed tomography (EBCT).

**Fig 6:**
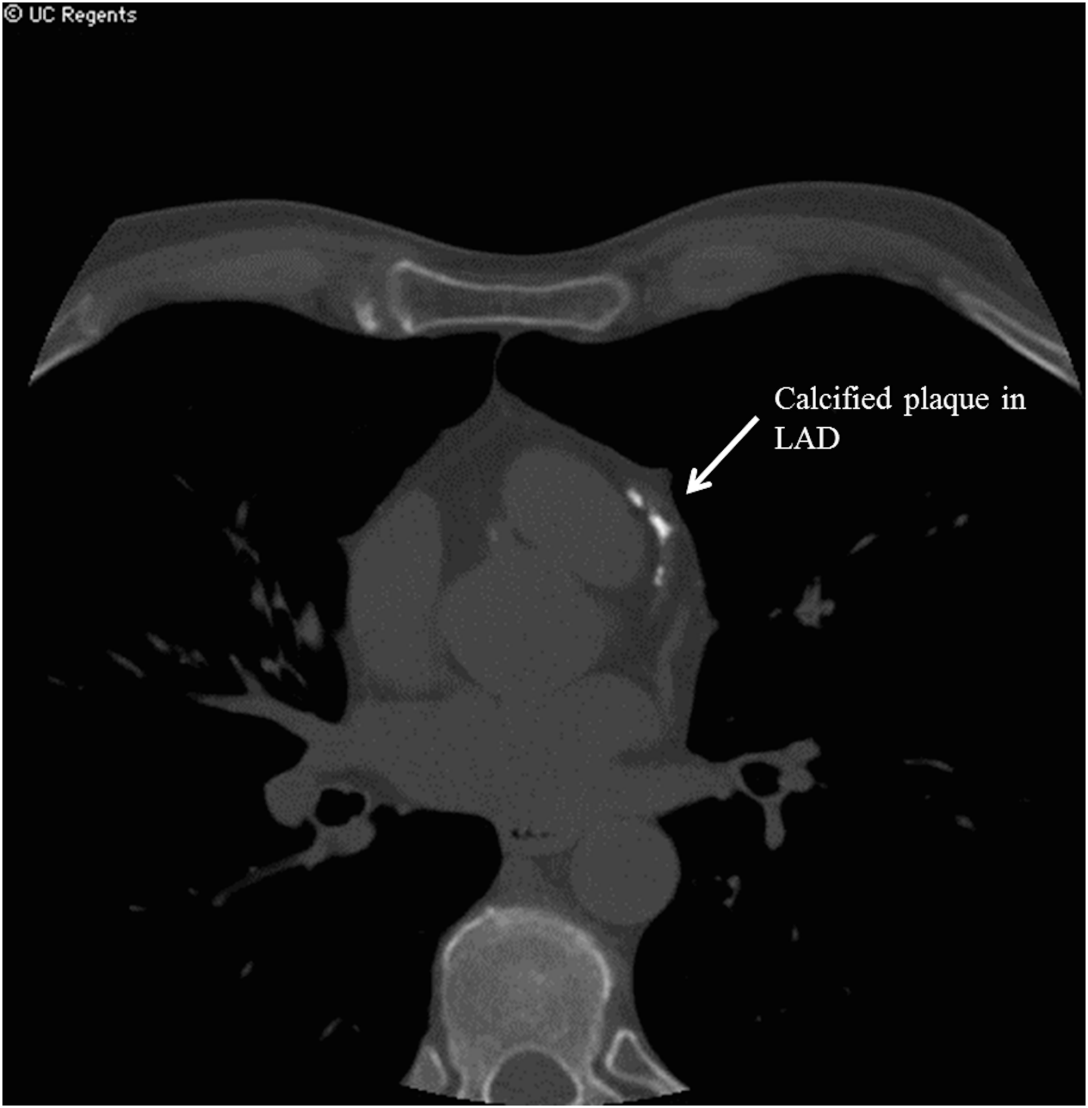
CAC EBCT of a subject with detectable CAC. Abbreviations: Coronary artery calcium (CAC), External beam computed tomography (EBCT).

Patients underwent CT imaging using a General Electric Light Speed\ 64-multi detector CT (GE Healthcare, Milwaukee, Wisconsin). Following the Agatston protocol, images were obtained in 2.5mm slices with no gap. Prospective cardiac gating was used to minimize motion artifact. Post-processing was performed in a dedicated work-station (Advanced Workstation Version 4.4, GE Healthcare, Milwaukee, Wisconsin). The detection of coronary calcium and quantitative calcium scores were calculated according to previously described methods (see Figs 5 and 6) [32]. A single cardiac radiologist (K.O.) performed the post-processing and interpretation of the scans and was blinded to all clinical characteristics of the subjects.

### Mortality Data

All mortality data were obtained from quarterly query of the National Death Index (NDI) and Social Security Death Index (SSDI). If subjects were not found within one of these two indices, they were presumed to be alive at the time of query.

### Statistical Analyses

Differences between the groups were assessed using the Student’s t-test. For continuous variables with markedly non-normal distributions, the Wilcoxon rank sum test was used. Categorical variables were compared with the Pearson’s χ^2^ test. Spearman rank correlation summarized associations between continuous variables. Multivariable linear regression analyses were performed to find independent predictors of increased age- and gender-adjusted CAC percentiles. Variables included in the model were age; gender; race; HIV-disease duration; CD4 count; CD4-nadir; HIV viral-load; history of HAART exposure; history of hepatitis C infection; hypertension; dyslipidemia; family history of coronary artery disease; tobacco pack-years; diabetes mellitus; the presence of MAC; prior CVD; current intravenous drug use and history of intravenous drug use. Survival curves were generated by the method of Kaplan and Meier and the Harrell’s C-statistic log rank test compared survival between groups. All tests were performed using a two-tailed level of significance of p < 0.05, using STATA software (StataCorp LP, College Station, Texas).

## Results

### Baseline Characteristics (entire cohort)

One-hundred fifty-two patients were enrolled in the study. Subjects’ median age was 50 years (Interquartile Range (IQR) 44 to 55 years). One hundred thirty two subjects (86.8%) were male. The median duration of HIV infection was 16 years (IQR 11 to 19 years), 127 subjects were on antiretroviral therapy (83.7%), and 97 subjects (64%) had an undetectable viral load. CVD risk factors included hypertension (35%), smoking (62%) and dyslipidemia (35%). Baseline characteristics for the cohort are displayed in Table 1.

**Table 1:** Baseline Characteristics—Entire Study Cohort.

Age (median years, IQR)	50 (IQR 44 to 55)
Female	20 (13%)
Hypertension	56(37%)
Dyslipidemia	53 (35%)
Diabetes Mellitus	15 (10%)
CAD	12 (8%)
History of MI	5 (3%)
Tobacco use	94 (62%)
Tobacco Pack-years (median pack-years, IQR)	5.7 (IQR 0 to 20.75)
Family History of CAD	33 (22%)
BMI (median BMI in kg/m^2^, IQR)	25.79 (IQR 23.33 to 29.28)
Baseline Creatinine (median mg/dL, IQR)	1.0 (IQR 0.88 to 1.13)
HIV duration (median years since diagnosis, IQR)	16 (IQR 11 to 19)
HIV RNA Level (median copies/mL, IQR)	75 (IQR 75 to 444)
CD4 Count (median cell/mL, IQR)	464 (IQR 288 to 658)
CD4 Count Nadir (median cell/mL, IQR)	120 (IQR 35 to 295)
History of OI	63 (41%)
History of HAART use	127 (84%)
HAART Duration (median years, IQR)	5.79 (IQR 0.75 to 7.83)
IVDU (current)	7 (5%)
IVDU (ever)	27 (18%)
HCV Infection	36 (24%)
CAC present	64 (42%)
MAC present	38 (25%)
MAC and CAC present	22 (14%)
Age- and Gender-adjusted CAC percentile (median percentile, IQR)	0.0 (IQR 0.0 to 62.5%)

Abbreviations: Interquartile Range (IQR), Coronary Artery Disease (CAD), Myocardial Infarction (MI), Human Immunodeficiency Virus (HIV), Human Immunodeficiency Virus Ribonucleic Acid Level (HIV RNA), Cluster of Differentiation 4 cells (CD4), Opportunistic Infection (OI), Highly Active Anti-Retroviral Therapy (HAART), Intravenous Drug-Use (IVDU), Hepatitis C Viral Infection (HCV), Coronary Artery Calcification (CAC), Mitral Annular Calcification (MAC)

### HIV-infected individuals with MAC

Thirty-eight (25%) of the 152 subjects had evidence of MAC on echocardiography. Baseline characteristics for subjects with and without MAC are shown in Table 2. There was no significant difference between the groups with regards to traditional cardiovascular risk factors (Table 2), except for family history of cardiovascular disease (CVD). Eight percent of HIV-infected individuals with MAC reported a family history of CVD, whereas 26% of those without evidence of MAC had a family history of CVD (p = 0.02). Median Body Mass Index (BMI) did not differ between the groups, and baseline creatinine levels were similar as well. There was no difference in duration of HIV infections (16 years IQR 10 to 19 vs. 16 years, IQR 11 to 19, p = 0.99), or in median HIV RNA level between the groups (75 copies/mL, IQR 75 to 5138 vs. 75 copies/mL, IQR 75 to 221, p = 0.07). Similarly, there was no significant difference in median CD4 count, median CD4 nadir, history of opportunistic infection, or duration of HAART use (p > 0.05 for all, Table 2). Current intravenous drug use was more common in the MAC group than in subjects without MAC (11% vs. 3%, p = 0.04); however, history of intravenous drug use did not differ between the groups (21% vs. 17%, p = 0.54), and there was no difference in Hepatitis C infection between the two groups.

**Table 2:** Baseline Characteristics—HIV-Subjects with MAC vs. those without MAC.

	MAC (n = 38)	No MAC (n = 114)	P value
Age (median years, IQR)	47.5 (IQR 42 to 54.25)	50 (IQR 44 to 55.25)	0.14^§^
Female (n, %)	3 (8%)	17 (15%)	0.30^‡^
Hypertension (n, %)	10 (26%)	46 (40%)	0.12^‡^
Dyslipidemia (n, %)	13 (34%)	40 (35%)	0.99^‡^
Diabetes Mellitus (n, %)	4 (11%)	11 (10%)	0.88^‡^
CAD (n, %)	5 (13%)	7 (6%)	0.17^‡^
History of MI (n, %)	1 (3%)	4 (4%)	0.79^‡^
Tobacco use (n, %)	22 (58%)	72 (63%)	0.56^‡^
Tobacco Pack-years (median Pack-years, IQR)	7.75 (IQR 0 to 20.75)	5.2 (IQR 0 to 21.38)	0.96^§^
**Family History of CAD (n, %)**	**3 (8%)**	**30 (26%)**	**0.02** ^**‡**^
BMI (median kg/m^2^, IQR)	24.73 (IQR 22.83 to 27.42)	26.18 (IQR 23.75 to 30.17)	0.10^§^
Baseline Creatinine (median mg/dL, IQR)	1.0 (IQR 0.83 to 1.16)	0.99 (IQR 0.90 to 1.10)	0.97^§^
HIV duration (median years since diagnosis, IQR)	16 (IQR 10 to 19)	16 (IQR 11 to 19)	0.99^§^
HIV RNA Level (median copies/mL, IQR)	75 (IQR 75 to 5138)	75 (IQR 75 to 221)	0.07^§^
CD4 Count (median cell/mL, IQR)	384.5 (IQR 210.3 to 618.8)	477 (IQR 297.5 to 622.5)	0.30^§^
CD4 Count Nadir (median cell/mL, IQR)	96 (IQR 33.75 to 218)	128 (IQR 35.5 to 300)	0.33^§^
History of OI (n, %)	20 (53%)	43 (38%)	0.11^‡^
History of HAART use (n, %)	34 (90%)	94 (83%)	0.30^‡^
HAART Duration (median years, IQR)	5.68 (IQR 1.89 to 8.08)	5.79 (IQR 0.33 to 7.81)	0.52^§^
**Current IVDU (n, %)**	**4 (11%)**	**3 (3%)**	**0.04** ^**‡**^
History of IVDU (n, %)	8 (21%)	19 (17%)	0.54^‡^
HCV Infection (n, %)	7 (18%)	29 (25%)	0.38^‡^
CAC detected (n, %)	21 (55%)	43 (38%)	0.09^‡^
**Age- and Gender-adjusted CAC percentile (median percentile, IQR)**	**44 (IQR 0 to 90)**	**0 (IQR 0 to 40)**	**0.01** ^**§**^

^§^Wilcox Rank-Sum Comparison P value

^‡^ Pearson’s χ^2^ Comparison P value

Abbreviations: Interquartile Range (IQR), Mitral Annular Calcification (MAC), Coronary Artery Disease (CAD), Myocardial Infarction (MI), Human Immunodeficiency Virus (HIV), Human Immunodeficiency Virus Ribonucleic Acid Level (HIV RNA), Cluster of Differentiation 4 cells (CD4), Opportunistic Infection (OI), Highly Active Anti-Retroviral Therapy (HAART), Intravenous Drug-Use (IVDU), Hepatitis C Viral Infection (HCV), Coronary Artery Calcification (CAC)

### Comparison of HIV-infected Subjects with and without CAC

Forty-two percent of the cohort had evidence of CAC on CT. Baseline characteristics for the patients who had CAC are shown in Table 3. Subjects who had CAC did not differ from those who did not have CAC with regards to gender, prevalence of hypertension, diabetes mellitus, history of MI, tobacco use or BMI. However, subjects with CAC were older (median age 53 years, IQR 48 to 59 vs. 46.5 years, IQR 42 to 51 years, p < 0.0001), had a higher rates of dyslipidemia (48% vs. 25%, p < 0.01) and coronary artery disease (16% vs. 2%, p < 0.01) when compared to subjects without CAC. HIV-disease characteristics including duration of HIV disease, HIV RNA, CD4 count, CD4 nadir, history of opportunistic infection, history of HAART use, duration of protease inhibitors, current abacavir use, and duration of HAART therapy did not differ between subjects with or without CAC. There was no difference in the prevalence of current or past intravenous drug use, and no difference in the prevalence of Hepatitis C viral infection between the two groups.

**Table 3:** Baseline Characteristics—Subjects with CAC vs. those without CAC.

	CAC (n = 64)	No CAC (n = 88)	P value
**Age (median years, IQR)**	**53 (IQR 48 to 59)**	**46.5 (IQR 42 to 51)**	**<0.0001** ^**§**^
Female (n, %)	5 (8%)	15 (17%)	0.23 ^‡^
Hypertension (n, %)	24 (38%)	32 (36%)	0.89 ^‡^
**Dyslipidemia (n, %)**	**31 (48%)**	**22 (25%)**	**0.003** ^**‡**^
Diabetes Mellitus (n, %)	8 (13%)	7 (8%)	0.35 ^‡^
**CAD (n, %)**	**10 (16%)**	**2 (2%)**	**0.003** ^**‡**^
History of MI (n, %)	4 (6%)	1 (1%)	0.08 ^‡^
Tobacco use (n, %)	39 (61%)	55 (63%)	0.85 ^‡^
Tobacco Pack-years (median Pack-years, IQR)	10 (IQR 0 to 23)	3.75 (IQR 0 to 20)	0.39^§^
Family History of CAD (n, %)	15 (23%)	18 (20%)	0.66 ^‡^
BMI (median kg/m^2^, IQR)	24.36 (IQR 22.56 to 28.91)	26.29 (IQR 23.95 to 29.53)	0.06^§^
HIV duration (median years since diagnosis, IQR)	16 (IQR 11 to 19)	15 (IQR 11 to 19)	0.45^§^
HIV RNA Level (median copies/mL, IQR)	75 (IQR 75 to 466)	75 (IQR 75 to 412)	0.55^§^
CD4 Count (median cell/mL, IQR)	476 (IQR 226.8 to 636.5)	456 (IQR 291 to 658)	0.99^§^
CD4 Count Nadir (median cell/mL, IQR)	128 (IQR 36 to 284)	110 (IQR 30 to 300)	0.99^§^
History of OI (n, %)	29 (45%)	34 (38%)	0.41 ^‡^
History of HAART use (n, %)	57 (89%)	71 (81%)	0.16 ^‡^
HAART Duration (median years, IQR)	5.84 (IQR 1.07 to 7.66)	5.65 (IQR 0.32 to 7.94)	0.57^§^
Current IVDU (n, %)	2 (3%)	5 (6%)	0.47 ^‡^
History of IVDU (n, %)	12 (19%)	15 (17%)	0.75 ^‡^
HCV Infection (n, %)	17 (27%)	19 (22%)	0.48 ^‡^
MAC detected (n, %)	21 (33%)	17 (19%)	0.09^‡^

^§^Wilcox Rank-Sum Comparison P value

^‡^ Pearson’s χ^2^ Comparison P value

Abbreviations: Interquartile Range (IQR), Coronary Artery Calcification (CAC), Coronary Artery Disease (CAD), Myocardial Infarction (MI), Human Immunodeficiency Virus (HIV), Human Immunodeficiency Virus Ribonucleic Acid Level (HIV RNA), Cluster of Differentiation 4 cells (CD4), Opportunistic Infection (OI), Highly Active Anti-Retroviral Therapy (HAART), Intravenous Drug-Use (IVDU), Hepatitis C Viral Infection (HCV), Mitral Annular Calcification (MAC)

### MAC and Coronary Calcium

**Fig 7:**
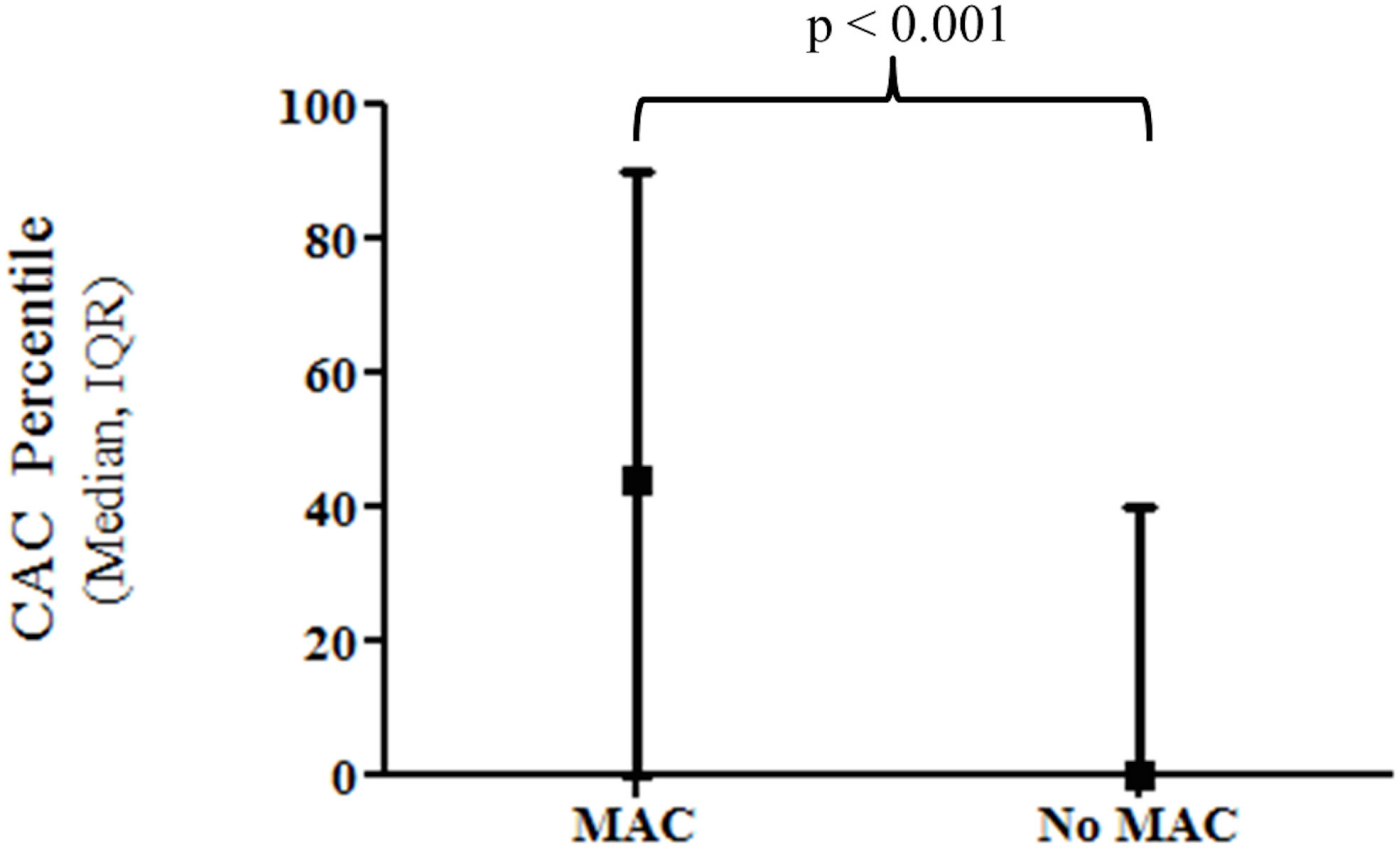
HIV-infected individuals with MAC have a higher median age- and gender-adjusted CAC percentile compared to those without MAC. Abbreviations: Coronary Artery Calcification (CAC), Mitral Annular Calcification (MAC), Interquartile Range (IQR).

The presence of MAC closely correlated with the presence of CAC, however, this trend did not reach statistical significance (OR 2.04, 95% CI 0.97 to 4.29, p = 0.06). The presence of MAC was associated with higher CAC scores. Namely, those with MAC had a median CAC score of 14.2 (IQR 0 to 150) versus a score of 0 (IQR 0 to 22) among those without MAC (p = 0.03). The presence of MAC was also associated with higher age- and gender-adjusted CAC percentiles (44% IQR 0 to 90% in MAC group vs. 0% IQR 0 to 40% in those without MAC, p < 0.001, Fig 7). Using echocardiography, M-mode measurements of MAC in the parasternal long axis views were significantly correlated with higher CAC composite scores (Spearman’s r 0.23, p = 0.005) and higher age- and gender-adjusted CAC percentiles (Spearman’s r 0.29, p = 0.001). In contrast, M-mode measurements of MAC performed in the apical four chamber view did not correlate with CAC composite scores or age- and gender-adjusted CAC percentiles (p = 1.0 and p = 0.84 respectively). After adjustment for HIV characteristics and traditional risk factors, the presence of MAC, prior CVD, older age and higher HIV RNA levels were independently associated with higher age- and gender-adjusted CAC percentiles (p < 0.05 for all).

### Mortality Data

**Fig 8:**
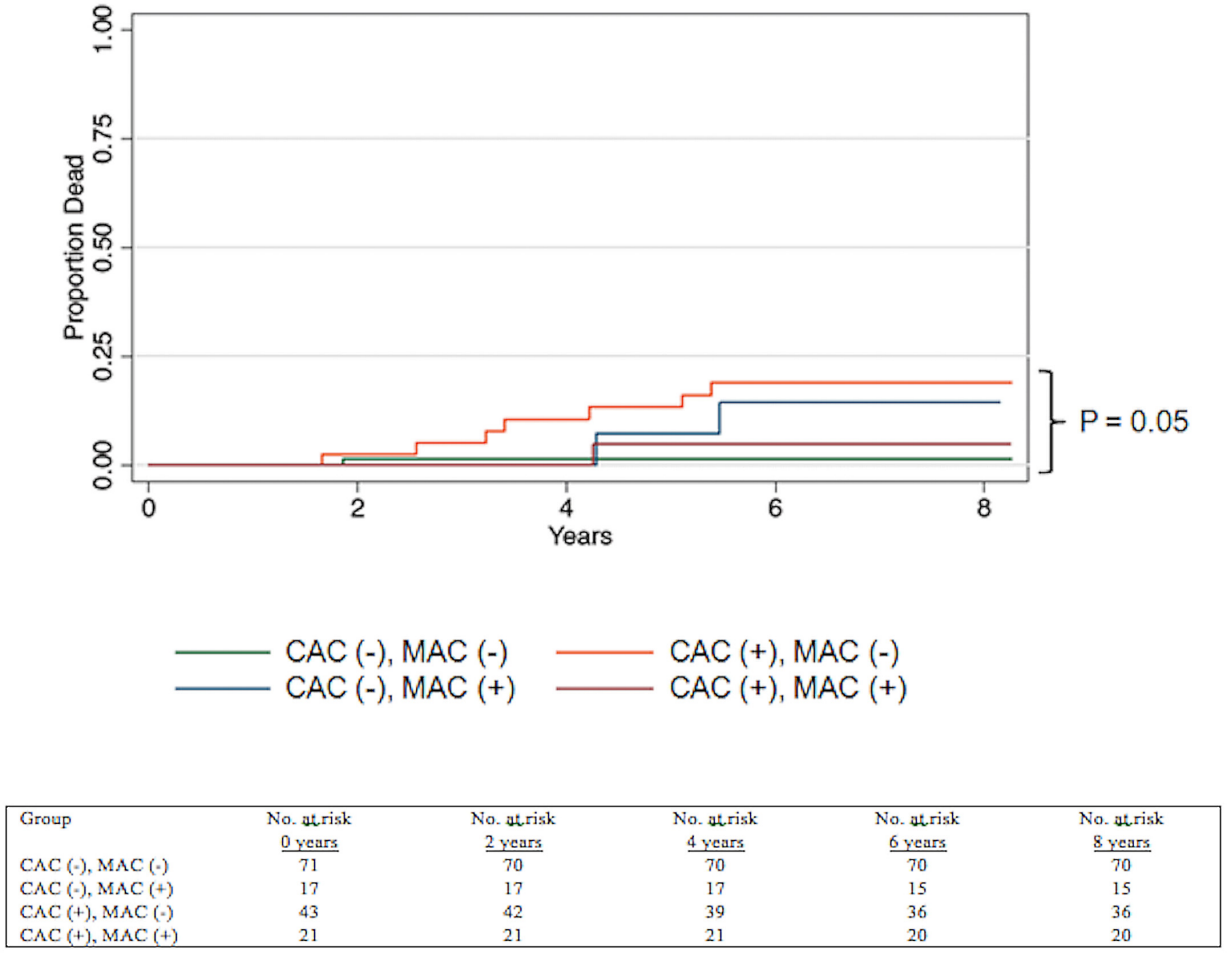
Kaplan Meier survival curve for subjects based on presence (+) or absence (-) of Coronary Artery Calcium, Mitral Annular Calcification. Abbreviations: Coronary Artery Calcification (CAC), Mitral Annular Calcification (MAC).

Over a median follow-up period of 8 years (IQR 7 to 8, 988 person-years), 11 subjects died (1.11 per 100 person-years). Seven of the eleven subjects who died had CAC without MAC (64%), two had MAC only (18%), and one had both MAC and CAC (9%). One of the eleven subjects who died had neither CAC nor MAC (9%). Subjects with CAC had significantly higher all-cause mortality compared to those without CAC, or compared to those with only MAC (2.88 per 100 person-years in the CAC group vs. 1.86 per 100 person-years in the MAC group vs. 0.20 per 100 person-years in the control group, p = 0.01, Fig 8). The Harrel’s C-statistic for predicting mortality was 0.66 for CAC alone and increased to 0.75 when MAC was added (comparison p = 0.05). The causes of death were variable, and included cardiac (n = 2, 18%), malignancy (n = 2, 18%), drug ingestion (n = 2, 18%), cirrhosis of the liver (n = 1, 9%), homicide (n = 1, 9%) and unknown (n = 3, 27%). Both cardiac deaths were in patients who had CAC on CT. We performed a sensitivity analysis and repeated our analysis after exclusion of individuals with known CAD. The findings of our sensitivity analysis were consistent with our findings in the entire cohort, namely, the Harrel’s C-statistic for predicting all-cause mortality was 0.66 for CAC alone and increased to 0.78 when MAC was added (comparison p = 0.03).

## Discussion

We demonstrate that MAC using echocardiography and CAC by CT scan are common among an asymptomatic cohort of HIV-infected individuals. The majority of these subjects were on anti-retroviral treatment and were virologically suppressed. The presence of CAC was associated with a significantly higher all-cause mortality rate among HIV-infected individuals when compared to those without CAC. The presence of MAC and CAC further increased the predictive value for all-cause mortality. To our knowledge this is one of the first reports to demonstrate the clinical significance of detectable CAC using EBCT in the HIV-positive patient population [33]. Our findings are consistent with previous studies of HIV-uninfected individuals which have demonstrated that the presence of CAC is predictive of major adverse cardiac events and mortality in asymptomatic individuals [28–32,34–38]. Nasir et al. studied a cohort of 44, 052 asymptomatic patients and found that individuals who had elevated CAC scores in the absence of traditional cardiovascular risk factors had significantly higher mortality rates than individuals with multiple risk factors but no CAC [28]. Of note, these patients were all referred for CAC screening based on clinician referral, and thus, the results are likely not applicable to the asymptomatic HIV-infected individuals we studied. Arad et al. followed 1,173 asymptomatic patients for an average of 19 months and found a consistent correlation between CAC and severity of CAD [37]. These patients were self-referred and outcome results were based largely on subject questionnaire response. Along with other studies, these findings have led to the growing popularity of using EBCT for identifying the presence of CAC. EBCT has now been incorporated into the current ACC/AHA guidelines to determine risk of future cardiovascular events among individuals at intermediate risk for cardiovascular disease or with a strong family history of cardiovascular disease [39]. While the utility of screening of all HIV patients using CAC remains ambiguous, our study demonstrates that detectable CAC along with MAC may identify a subset of HIV-infected individuals at higher risk for all-cause mortality.

There have been several studies which have examined the prevalence and severity of CAC in the HIV-infected population, but these studies report very few clinical outcomes [13,40,41]. Mangili et al. performed a cross-sectional analysis of 327 HIV-infected individuals who had undergone carotid artery intima-media thickness screening (cIMT) and CAC EBCT and found that for men included in the study, age, apolipoprotein B level, and high-sensitivity C-reactive protein level independently predicted CAC score. Age and glucose level independently predicted CAC score in women [40]. Guaraldi and colleagues performed a cross-sectional analysis of 400 HIV-infected individuals who had undergone CAC EBCT and found that 40.5% of the cohort had increased vascular age on EBCT, but again, no clinical outcomes were reported [42]. A multi-variate analysis revealed that current CD4 count was the only predictor of increased vascular age [13]. Fitch and colleagues found that HIV-infected individuals who had the metabolic syndrome, as well as HIV-infected individuals who did not have the metabolic syndrome, demonstrated an increased prevalence of calcified and non-calcified plaque segments on CAC EBCT when compared to non-infected controls. Recently, a large study from the MultiCenter Aids Clinical cohort (MACS), demonstrated that HIV features were independently associated with non-calcified plaque at baseline [43]. However, to our knowledge, none of these prior studies correlated CAC or other findings with mortality or other clinical outcomes in the setting of HIV infection.

The presence of MAC using echocardiography has not been previously described among individuals with HIV. A study following elderly individuals without HIV for 16 years demonstrated an independent association between MAC and incident cardiovascular disease and cardiovascular mortality [20]. Atar and colleagues reported that more than 1/3 subjects who were screened with TTE had evidence of MAC. In turn, the presence of MAC was associated with a higher prevalence of coronary artery disease, left main disease, and triple vessel disease as revealed by cardiac catheterization. The positive predictive value of MAC for severe coronary artery disease was 92% [15]. While echocardiographic studies performed in modern HIV cohorts have reported high rates of diastolic dysfunction and elevated pulmonary artery pressures [44], our report is the first to evaluate MAC in the setting of HIV infection and to link this echocardiographic finding to clinical outcomes along with CAC.

Cardiovascular disease is the second most common cause of non-AIDS deaths among individuals with HIV in the United States [45]. As the HIV patient population continues to age, HIV-associated cardiovascular complications will become an increasingly significant health issue. The risk for acute MI is more than doubled in the setting of HIV infection and this increased risk persists among treated and suppressed HIV-infected individuals [10]. Our group has previously described high rates of sudden cardiac death, one of the most deadliest manifestations of cardiovascular disease, among individuals with HIV [12]. Although the heightened risk of cardiovascular events and mortality in the HIV-population has been well studied, few studies have explored the utility of non-invasive studies to identify individuals at risk. As such, there is no consensus regarding the use of these non-invasive assessments of cardiovascular disease in HIV-infected subjects. Much of our current clinical practice is driven by extrapolation of findings from studies of HIV-negative subjects. The results of this study suggest that EBCT screening for CAC may be useful for identifying HIV-infected individuals at high risk for all-cause mortality. Furthermore, the addition of the presence of MAC to CAC resulted in significant improvement in the characteristics of the Harrel’s C-statistic for prediction of individuals at the highest risk for death over the course of this study (988 person-years). When quantified, MAC is typically labeled as mild, moderate or severe, and these gradations are highly subjective and have sub-optimal inter-observer variability. Our study demonstrated a linear correlation between MAC when measured in the parasternal long axis view and CAC score, as well as age- and gender-adjusted CAC percentile. This correlation did not hold when MAC was measured in the apical four chamber view, which may be due to measuring technique or artifact when measured in this view. These findings are relevant as they suggest there is a quantifiable relationship between MAC and CAC, and they have biological plausibility as calcification is likely a systemic process affecting both the heart valves and coronaries at the same time.

Previous studies have also demonstrated that age- and gender-adjusted CAC percentile is the preferred parameter for predicting cardiovascular and mortality outcomes using CAC-protocol EBCT data [34,35,38]. Population based studies have demonstrated the overall prevalence of MAC to be roughly 13%^44^ and CAC to be between 40–60% in subjects with a mean age of 53 ± 11 years [34,37,46,47]. We found a similar prevalence of CAC in our HIV-infected individuals along with a markedly higher prevalence of MAC despite the younger age of our cohort (mean age 49.4 ± 0.7 years). Post and colleagues demonstrated coronary artery plaque, particularly non-calcified plaque is more prevalent and extensive in HIV-infected men, independent of CAD risk factors, and thus future studies will be needed to ascertain if non-calcified plaque may be more predictive of events as compared to CAC in HIV-infected individuals. As our study only examined coronary artery calcification, we were not able to evaluate non-calcified plaques.

The findings are consistent with previously proposed theories of an accelerated vascular aging complex related to the HIV virus [13,48]. In this study, after adjustment for HIV characteristics and traditional risk factors, including age, gender, tobacco use, hypertension, dyslipidemia, and diabetes mellitus, the presence of MAC, prior CVD, older age and higher HIV RNA levels were independently associated with higher age- and gender-adjusted CAC percentiles. While age is usually the strongest predictor of calcium, our findings suggest that uncontrolled HIV disease may also contribute to coronary calcification and thus increased cardiovascular risk.

### Limitations

There are several limitations to this study. Since this is a retrospective cohort, we cannot assume or invoke causality for any of the associations that were found. Our study evaluated the predictive value of baseline prevalent MAC and CAC upon mortality in HIV; future studies which incorporate serial imaging will be needed to evaluate the impact of incidence of MAC or CAC on mortality. The sample size was also small and may be under-powered to determine differences in mortality outcomes, particularly cardiovascular death, as there were very few of these events during the course of the study. This study was performed at a single center, thus, the population studied may not be representative of the HIV-positive population as a whole. Some of the variables studied, including tobacco use, pack-years, family history of coronary artery disease and past or present injection drug use relied on subject self-report, and thus may be susceptible to reporting bias; however it is unlikely that there was differential reporting bias among those with and without CAC and/or MAC. As the baseline demographic variables and clinical outcome variables (death) were obtained via query of existing databases, we were only able to report variables which were available in the respective databases. Other outcomes such as new cardiovascular or coronary events were not available at the time of query. Finally, the Agatston scoring method for coronary artery calcification has several limitations, which have been well documented. These include image noise and artifact influencing CAC score, the non-linear increases in calcium score due to density numbers used to categorize pixel intensity, and the inaccuracies in the Agatston score, which may under- or over-estimate the true volume of calcium in a given lesion [49].

## Conclusions

The current study demonstrates that 1) MAC and CAC among HIV-infected individuals are common, 2) the presence of both CAC and MAC identify HIV-positive patients at elevated risk for all-cause mortality, 3) there is a significant association between mitral annular calcification and age- and gender-adjusted coronary calcium percentile, and 4) traditional risk factors along with HIV viral load are independently associated with CAC. Taken together, these findings support the premise that HIV infection may play a role in accelerated vascular and cellular aging and that presence of systemic calcium may identify HIV-infected individuals at high risk for all-cause mortality. Further investigation is needed to determine whether modification of cardiovascular risk factors and / or HIV disease activity can alter HIV-positive subject’s coronary artery or mitral annular calcification profile.

## Supporting Information

S1 Table Microsoft Excel Database. Attached please find our anonymous primary dataset. The most pertinent portions of this dataset include sheet 1, titled, “MAC HIV subjects, no controls” and sheet 2, titled, “MAC Dataset Legend.” Sheet 1 is titled as such because all subjects in this database have a diagnosis of HIV. HIV-negative control subjects, which are found in the SCOPE database, were not included in this study. Sheet 1 has all primary information that was obtained via chart review, primary review of the two-dimensional transthoracic echocardiograms and computed tomography scans as described in the methods section. Sheet 2 has more detailed descriptions of the variables that were recorded as well as translation codes.
(XLS)
